# Predictive Value of Arterial Enhancement Fraction Derived from Dual-Layer Spectral Computed Tomography for Thyroid Microcarcinoma

**DOI:** 10.3390/diagnostics15192427

**Published:** 2025-09-23

**Authors:** Yuwei Chen, Jiayi Yu, Liang Lv, Zuhua Song, Jie Huang, Bi Zhou, Xinghong Zou, Ya Zou, Dan Zhang

**Affiliations:** 1 Department of Radiology, Chongqing General Hospital, Chongqing University, Chongqing 401147, China; chenyuwei1999@cqu.edu.cn (Y.C.); yujiayi511@sohu.com (J.Y.); lvliang506@sohu.com (L.L.); szuh2013@foxmail.com (Z.S.); hj5332@foxmail.com (J.H.); zhoubifangshe@foxmail.com (B.Z.); 15683834881@sohu.com (X.Z.); 130546@cqu.edu.cn (Y.Z.); 2 Department of Radiology, The Second Affiliated Hospital of Chongqing Medical University, Chongqing 400010, China

**Keywords:** thyroid nodule, dual-layer spectral detector CT, arterial enhancement fraction, diagnosis, differential

## Abstract

**Background/Objectives:** Accurately distinguishing malignancy in thyroid micronodules (≤10 mm) is crucial for clinical management, yet it is challenging due to the limitations of conventional ultrasonography-guided biopsy. This study aims to evaluate the predictive value of dual-layer spectral computed tomography (DSCT)-derived arterial enhancement fraction (AEF) in diagnosing thyroid microcarcinomas. **Methods:** In the study, 321 pathologically confirmed thyroid micronodules (benign = 131, malignant = 190) from Chongqing General Hospital underwent preoperative DSCT. Quantitative parameters of DSCT, including the normalized iodine concentration (NIC), normalized effective atomic number (NZ_eff_), and slope of the spectral Hounsfield unit curve (*λ*HU_(40–100)_), were assessed. Both single-energy CT (SECT)-derived AEF (AEF_S_) and DSCT-derived AEF (AEF_D_) were calculated. Conventional image features included microcalcifications and enhancement blurring. Correlation between AEF_D_ and AEF_S_ was determined using Spearman’s correlation coefficient. Diagnostic performance was evaluated by calculating the area under the curve (AUC) using receiver operating characteristic (ROC) analysis. **Results:** Malignant micronodules exhibited significantly lower AEF_D_ (0.958 vs. 1.259, *p* < 0.001) and AEF_S_ (0.964 vs. 1.436, *p* < 0.001) versus benign nodules. Arterial phase parameters—AP*λ*HU_(40–100)_, APNIC, APNZ_eff_—differed significantly between groups (all *p* < 0.001), whereas venous phase parameters (VP*λ*HU_(40–100)_, VPNIC, VPNZ_eff_) showed no differences (all *p* > 0.05). Multivariate analysis revealed that *λ*HU_(40–100)_ as an independent predictor of malignancy, with an odds ratio (OR) of 0.600 (95% confidence interval (CI): 0.437–0.823; *p* = 0.002) and an AUC of 0.752 (95% CI: 0.698–0.806). A significant positive correlation was identified between AEF_D_ and AEF_S_ (*r* = 0.710; *p* < 0.001). For diagnosing malignancy, AEF_D_ demonstrated superior overall performance (AUC: 0.794; sensitivity: 70.5%; specificity: 81.7%; accuracy: 75.1%) to AEF_S_ (0.753; 71.1%; 74.0%; 72.3%), AP*λ*HU_(40–100)_ (0.752; 68.9%; 75.6%; 71.7%), and calcification (0.573; 21.6%; 92.4%; 50.5%). Clinically, AEF_D_ reduced the unnecessary biopsy rate to 18.3%, preventing 107 procedures in our cohort. **Conclusions:** AEF_D_ and AEF_S_ demonstrated strong correlation and comparable diagnostic performance in the evaluation of thyroid micronodules. Furthermore, AEF_D_ showed favorable diagnostic efficacy compared to both spectral parameters and conventional imaging feature. More importantly, the application of AEF_D_ significantly reduced unnecessary biopsy rates, highlighting its clinical value in optimizing patient management.

## 1. Introduction

The rising incidence of thyroid cancer, especially in small nodules, has caused significant concern among medical professionals [1,2]. Thyroid microcarcinomas (TMCs), defined as cancerous nodules less than 1 cm in diameter, pose diagnostic challenges due to their often indolent nature [3,4,5]. Misdiagnosis or indeterminate findings can lead to suboptimal management. False-negative results may delay treatment of malignancies, which can lead to disease progression [6]. Conversely, false-positive findings or over-diagnosis frequently lead to unnecessary thyroid surgery, carrying potential complications, such as permanent hypothyroidism and recurrent laryngeal nerve injury [7,8]. These interventions contribute to patient distress and impose a substantial financial burden on healthcare systems through unnecessary procedures and long-term follow-up [9,10]. Current management of TMCs includes both active surveillance and surgical intervention, both demonstrating comparable 20-year survival outcomes [11]. Benign micronodules require no intervention. Thus, accurate discrimination between benign and malignant micronodules is critical for guiding clinical decision-making.

Ultrasound is the first-line imaging modality for thyroid nodules due to its high resolution, lack of radiation, low cost, and real-time capability, allowing for detailed morphological assessment [12]. However, it is operator-dependent and suffers from inter-observer variability, with limited utility for mediastinal ectopic glands [13]. Fine-needle aspiration cytology (FNAC) remains the gold standard for pathological diagnosis, particularly for sonographically suspicious nodules [14], yet it is invasive—carrying risks of bleeding or infection [15]—and may yield indeterminate or false-negative results [16,17]. Elastography offers objective quantification of tissue stiffness, complementing conventional ultrasound with high sensitivity and specificity [18,19], though its accuracy is influenced by nodule heterogeneity and operator experience [20]. While ^F^18-fludeoxyglucose positron emission tomography (^F^18-FDG-PET) can accurately predict benign pathology [21], it is not recommended for initial evaluation of thyroid nodules or indeterminate cytology due to high cost, limited availability, and lack of standardization [13,22]. Magnetic resonance imaging (MRI) provides excellent soft-tissue resolution without ionizing radiation, but is restricted by cost, acquisition time, and motion artifacts; it is mainly reserved for evaluating extrathyroidal extension [23]. Conventional computed tomography (CT) offers three-dimensional cervical anatomy with high accessibility and rapid imaging [24], but its accuracy for microcarcinomas is limited by poor lesion contrast, overlapping features with benign micronodules, and artifacts [17,25]. Thus, there is a critical need to develop cost-effective, non-invasive techniques that provide objective quantitative data for accurately differentiating thyroid micronodules.

Dual-layer spectral computed tomography (DSCT) simultaneously acquires high- and low- energy data, enabling material decomposition for quantitative measures, such as iodine concentration (IC) and effective atomic number (Z_eff_) [26], which has demonstrated improved diagnostic performance in differentiating thyroid micronodules beyond conventional radiological features [27]. Furthermore, the arterial enhancement fraction (AEF) derived from dual-energy CT has proven valuable in detecting cervical lymph node metastasis in papillary thyroid carcinoma, suggesting that DSCT-derived AEF (AEF_D_) may also aid in distinguishing TMCs from benign micronodules [28]. Nevertheless, significant knowledge gaps persist. Current imaging techniques remain insufficient for fully characterizing the biological behavior of TMCs. The most reliable independent predictors among DSCT quantitative parameters have not been established, and conventional features fail to elucidate underlying pathophysiology, underscoring the need for molecular biomarkers. Moreover, the diagnostic performance of AEF_D_ relative to other quantitative parameters and imaging features remains unexplored.

The specific objectives of this research are to assess the clinical utility of the AEF_D_ and its potential to refine workflows for thyroid nodules. By comparing the efficacy of AEF_D_ with both advanced quantitative parameters and traditional radiological, this study seeks to provide an evidence-based framework for improving diagnostic outcomes and, ultimately, patient care in the context of thyroid disease management. This work is anticipated to contribute to ongoing efforts in optimizing diagnostic and therapeutic strategies for thyroid cancer, addressing the pressing need for more reliable differentiation between malignant and benign micronodules.

## 2. Materials and Methods

### 2.1. Patient Cohort

**Figure 1:**
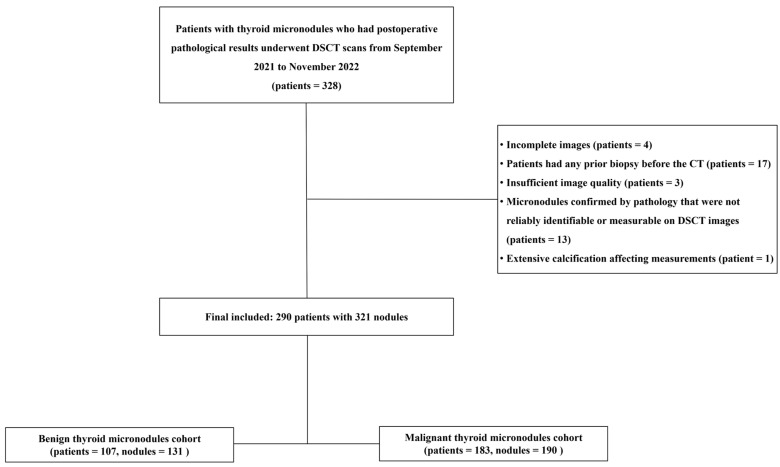
Flow diagram of the study cohort selection for thyroid micronodules. DSCT = Dual-layer spectral detector computed tomography; CT = computed tomography.

This retrospective study was approved by the Medical Ethics Committee of Chongqing General Hospital with a waiver of informed consent. Preliminary preoperative DSCT data from 328 patients were collected between September 2021 and November 2022 from a picture archiving and communication system (Vue PACS Version 3. 2. 0501. 0, Philips Healthcare, Amsterdam, The Netherlands). Diagnosis of all micronodules was confirmed by postoperative pathology. The exclusion criteria were as follows: (1) incomplete imaging data; (2) previous biopsy prior to DSCT; (3) significant artifacts or noise affecting image quality; (4) micronodules not reliably identifiable or measurable on DSCT relative to pathological findings; (5) extensive calcification preaccurate measurement. Ultimately, 290 patients with 321 thyroid micronodules (190 malignant, 131 benign) were included. The flowchart of patient selection is shown in Figure 1.

### 2.2. DSCT Image Acquisition

All participants in the study underwent neck CT examinations on a 64-slice dual-layer spectral CT scanner (IQon Spectral CT, Philips Healthcare, Amsterdam, The Netherlands), including non-contrast and contrast-enhanced scans. The acquisition protocol included the following parameters: a tube voltage of 120 kV; tube current modulated by an automated exposure control system (DoseRight, Philips Healthcare); a detector collimation of 64 × 0.625 mm; a field of view (FOV) of 350 mm; a matrix size of 512 × 512; a layer thickness of 5 mm, and a reconstruction thickness of 1.25 mm. After the non-contrast CT scanning, contrast-enhanced CT was performed using bolus-tracking with a region of interest (ROI) placed in the descending aorta at the tracheal bifurcation. Non-ionic contrast (1.5 mL/kg) was injected at a rate of 3.5 mL/s, immediately followed by a 30 mL saline flush to ensure proper distribution. The arterial phase was triggered 6 s after reaching a threshold of 150 Hounsfield units (HUs) within the ROI, and the venous phase was acquired 40 s after arterial phase initiation.

### 2.3. Nodule Matching and Selection Criteria

Nodules were matched according to the following criteria: (1) nodule location (left lobe, right lobe, or isthmus and superior, middle, or inferior) was determined from pathological reports; (2) each nodule was identified on CT using its location and size; (3) for clustered nodules, the largest per pathology was selected. Unmatched nodules were excluded.

### 2.4. Qualitative Image Analyses

Two radiologists, one with 7 years and the other with 4 years of experience in head and neck radiology, conducted a qualitative assessment of the CT image features. They were blinded to the study design and final results. All analyses were performed solely on non-contrast, arterial phase (AP), and venous phase (VP) CT images. Disagreements were resolved by a third senior radiologist who had 17 years of experience in head and neck imaging. Analyzed features included microcalcification (calcific foci ≤ 2 mm in diameter) and enhanced attenuation blurring, characterized by reduced nodule–thyroid interface demarcation and decreased attenuation difference relative to the surrounding parenchyma post-contrast.

### 2.5. Quantitative Measurements of Spectral Parameters

Quantitative analysis of the AP and VP images were performed using a specialized spectral CT post-processing workstation (IntelliSpace Portal Version 10.1, Philips Healthcare, Amsterdam, The Netherlands). Reconstruction datasets included monoenergetic maps (40–100 keV), iodine density maps, and effective atomic number maps. ROIs were manually delineated to cover approximately two-thirds of each micronodule’s cross-sectional area while carefully excluding necrosis, cystic degeneration, and calcifications. Furthermore, a reference ROI was positioned within the core area of the carotid artery at the corresponding level. The ROIs’ location, shape, and size were maintained constant across different phases through the use of the copy-and-paste function.

The following parameters were automatically calculated for our study: HU values of micronodules at 40 keV, 70 keV, and 100 keV monoenergetic levels in both AP and VP, designated as AP_40keV_, AP_70keV_, AP_100keV_, VP_40keV_, VP_70keV_, and VP_100keV_. Additionally, the slope of the spectral Hounsfield unit curve (*λ*HU) for each phase was calculated as follows:

(1) $$\lambda \mathrm{HU}=\frac{{\mathrm{HU}}_{40\mathrm{keV}}-{\mathrm{HU}}_{100\mathrm{keV}}}{100-40}$$

Iodine concentration (IC) and effective atomic number (Z_eff_) were measured directly from iodine density maps and effective atomic number maps, respectively. To account for inter-scan variability and enhance comparability, both IC and Z_eff_ values were normalized to the values measured from the carotid artery during in the same phase. This normalization yielded the parameters of the normalized iodine concentration (NIC) and normalized effective atomic number (NZ_eff_). The formulas for normalization are as follows:

(2) $$\mathrm{NIC}\;=\;\frac{\mathrm{Nodule}\;\mathrm{IC}}{\mathrm{Carotid}\;\mathrm{artery}\;\mathrm{IC}},$$

(3) $${\mathrm{NZ}}_{\mathrm{eff}}=\frac{\mathrm{Nodule}\;Z_{\mathrm{eff}}}{\mathrm{Carotid}\;\mathrm{artery}\;Z_{\mathrm{eff}}},$$

where Nodule IC and Nodule Z_eff_ represent the iodine concentration and effective atomic number within the thyroid nodule, respectively, and Carotid artery IC and Carotid artery Z_eff_ represent the corresponding values within the carotid artery. These normalized parameters were calculated separately for the AP and VP scans as APNIC, VPNIC, APNZ_eff_, and VPNZ_eff_.

### 2.6. Quantitative Measurements of AEF_D_ and AEF_S_

**Figure 2:**
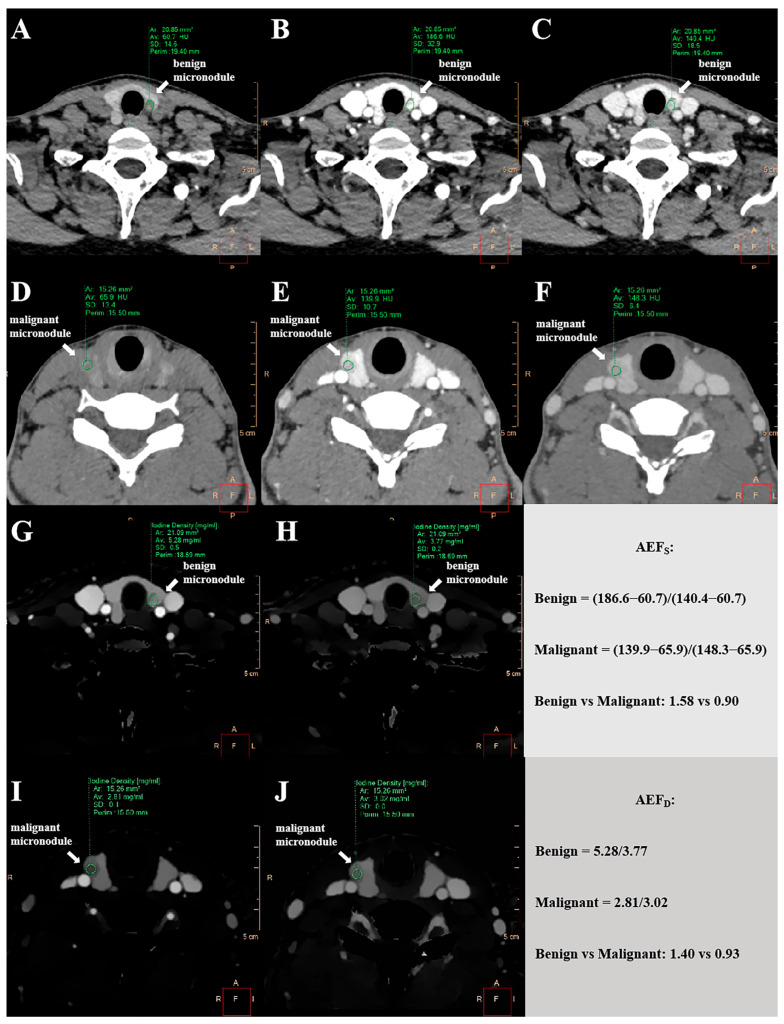
Comparison of AEF between malignant and benign thyroid micronodules. (**A**–**C**) AEF_S_ measurement in a 50-year-old female patient with a benign micronodule (arrow). CT values of non-contrast phase (**A**), arterial phase (**B**), and venous phase (**C**) on axial conventional CT images were 60.70 HU, 186.60 HU, and 140.40 HU, respectively. AEF_S_ calculated as (HU_a_ − HU_u_)/(HU_v_ − HU_u_). (**D**–**F**) AEF_S_ measurement in a 30-year-old female patient with a malignant micronodule (arrow). CT values of non-contrast phase (**D**), arterial phase (**E**), and venous phase (**F**) on axial conventional CT images were 65.90 HU, 139.90 HU, and 148.30 HU, respectively. AEF_S_ calculated as (HU_a_ − HU_u_)/(HU_v_ − HU_u_). (**G**,**H**) AEF_D_ measurement in the same benign micronodule (arrow) on axial iodine maps derived from spectral-based imaging. IC values of arterial phase (**G**) and venous phase (**H**) were 5.28 mg/mL and 3.77 mg/mL. AEF_D_ calculated as (IC_a_)/(IC_v_). (**I**,**J**) AEF_D_ measurement in the same malignant micronodule (arrow) on axial iodine maps derived from spectral-based imaging. IC values of arterial phase (**I**) and venous phase (**J**) were 2.81 mg/mL, 3.02 mg/mL. AEF_D_ calculated as (IC_a_)/(IC_v_). The AEF_S_ (0.90 vs. 1.58) and AEF_D_ (0.93 vs. 1.40) of the malignant micronodule were lower than those of the benign micronodule. AEF = arterial enhancement fraction; AEF_S_ = single-energy computed tomography-derived arterial enhancement fraction; AEF_D_ = dual-layer computed tomography-derived arterial enhancement fraction; CT = computed tomography; HU = Hounsfield unit; IC = iodine concentration.

The AEF_S_ was measured using a 120-kVp equivalent blended CT image, which included the non-contrast (HU_u_), arterial (HU_a_), and venous phases (HU_v_), calculated as follows:

(4) $${\mathrm{AEF}}_{S}\;=\;\frac{{\mathrm{HU}}_{a}-{\mathrm{HU}}_{u}}{{\mathrm{HU}}_{v}-{\mathrm{HU}}_{u}},$$

where HU_u_, HU_a_, and HU_v_ represent the HU values within the thyroid nodule in the non-contrast, arterial, and venous phases, respectively. The AEF_D_ was calculated using IC measurements from iodine maps during the arterial (IC_a_) and venous phases (IC_v_), defined as follows:

(5) $${\mathrm{AEF}}_{D}\;=\;\frac{{\mathrm{IC}}_{a}}{{\mathrm{IC}}_{v}}$$

where IC_a_ and IC_v_ represent the iodine concentration within the thyroid nodule in the arterial and venous phases, respectively. All measurements were made using manually placed ROIs carefully avoiding areas of calcification, cysts, and necrosis. Figure 2 provides a schematic illustration of the quantitative measurements for both the AEF_D_ and AEF_S_.

### 2.7. Statistical Analyses

Statistical analyses were performed using SPSS (IBM Corp., Version 27.0, Chicago, IL, USA). The normality of continuous variables was assessed using the Kolmogorov–Smirnov test. Variables normally distributed with homogeneous variances were compared using the independent samples *t*-test; otherwise, the Mann–Whitney *U* test was applied. Categorical variables were compared using the *χ*^2^ test or Fisher’s exact test, as appropriate. The correlation between AEF_D_ and AEF_S_ was determined using Spearman’s rank correlation coefficient. Agreement between these two measurements was visualized using Bland–Altman plots, with systematic bias and limits of agreement calculated. Multivariate logistic regression analysis was performed using the forward variable selection method. Diagnostic performance was evaluated by receiver operating characteristic (ROC) curve analysis, with the optimal cutoff determined by maximizing Youden’s index. A *p*-value < 0.05 was considered statistically significant.

## 3. Results

### 3.1. Comparative Analysis of Demographic and DSCT Parameters in Benign Versus Malignant Thyroid Micronodules

A total of 321 thyroid micronodules in 290 patients (131 benign and 190 malignant) were included in this study. In the benign cohort, 44.9% of patients (48 out of 107) were aged 50 years or older, while 55.1% (59 out of 107) were younger than 50 years. In contrast, the malignant cohort had 14.2% (26 out of 183) aged 50 years or more and 85.8% (157 out of 183) younger than 50 years (*p* < 0.001). The gender distribution revealed that 91.6% (98 out of 107) of the benign cohort were female, compared to 84.2% (154 out of 183) of the malignant cohort (*p* = 0.070). Calcification patterns differed significantly: the benign cohort exhibited absent calcifications in 92.4% (121/131), microcalcifications in 2.3% (3/131), and macrocalcifications in 5.3% (7/131); the malignant cohort showed absent calcifications in 78.4% (149/190), microcalcifications in 15.8% (30/190), and macrocalcifications in 5.8% (11/190) (*p* < 0.001). The maximum diameters of nodules were 6 mm (interquartile range (IQR): 5–9) in benign cohort versus 7 mm (IQR: 6–8) in malignant cohort (*p* = 0.392). Enhanced blurring was observed in 32.8% (45/131) of the benign cohort and 65.3% (124/190) of the malignant cohort (*p* = 0.722). Venous phase parameters showed no statistical differences between the benign and malignant cohorts, including VP*λ*HU_(40–100)_ (*p* = 0.942), VPNIC (*p* = 0.194), and VPNZ_eff_ (*p* = 0.103). In contrast, arterial phase parameters demonstrated group differences for AP*λ*HU_(40–100)_ (*p* < 0.001), APNIC (*p* < 0.001), and APNZ_eff_ (*p* < 0.001) (Table 1).

The multivariate logistic regression analysis yielded the following results: for AP*λ*HU _(40–100)_, regression coefficient (*β*) = −0.511 and odds ratio (OR) = 0.600 (95% confidence interval (CI): 0.437–0.823, *p* = 0.002); for APNIC, *β* = −4.589 and OR = 0.010 (95% CI: 0.000–4.658, *p* = 0.142); and for APNZ_eff_: *β* = −0.883 and OR = 0.414 (95% CI: 0.000–4613.544, *p* = 0.853). The model explained 17.7% of the variance (standardized *R*^2^ = 0.177, *F*–statistic = 22.752) with all variance inflation factors below 5 (Table 2).

### 3.2. Comparison of AEF Values Between Benign and Malignant Thyroid Micronodules

**Figure 3:**
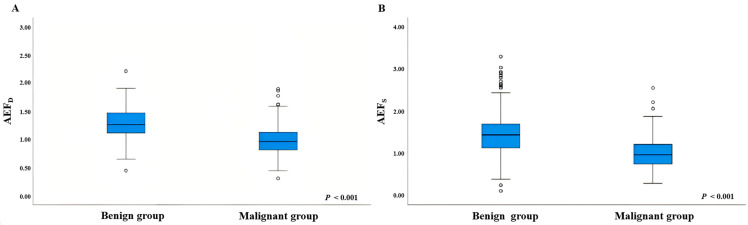
Box plots comparing the values distribution of (**A**) dual-layer detector CT-derived arterial enhancement fraction (AEF_D_) and (**B**) single-energy CT-derived arterial enhancement fraction (AEF_S_) between benign and malignant thyroid micronodule cohorts. The central line in each box represents the median, the box extends to the interquartile range (IQR), and the whiskers show the range. The median AEF_D_ and AEF_S_ values were significantly lower in malignant nodules than in benign nodules (both *p* < 0.001).

Benign nodules exhibited a median AEF_S_ of 1.436 (IQR 1.126–1.697), while malignant nodules had a median AEF_S_ of 0.964 (IQR 0.747–1.210) (*p* < 0.001). The median AEF_D_ was 1.259 (IQR 1.112–1.469) in benign versus 0.958 (IQR 0.811–1.123) in malignant nodules (*p* < 0.001). Parameter distributions are shown in Figure 3, with detailed data in Table 3.

### 3.3. Correlation Between AEF_D_ and AEF_S_

**Figure 4:**
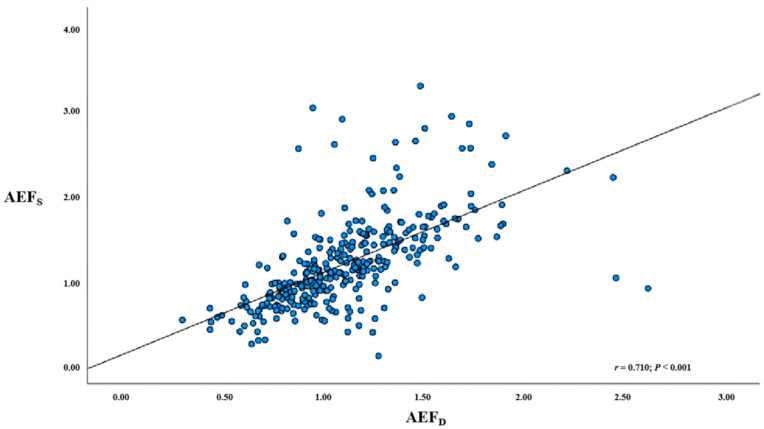
A scatterplot of correlation between AEF_S_ and AEF_D_; a significant positive linear correlation was observed (*r* = 0.710; *p* < 0.001). AEF_D_ = dual-layer computed tomography-derived arterial enhancement fraction; AEF_S_ = single-energy computed tomography-derived arterial enhancement fraction; *r* = Pearson correlation coefficient.

**Figure 5:**
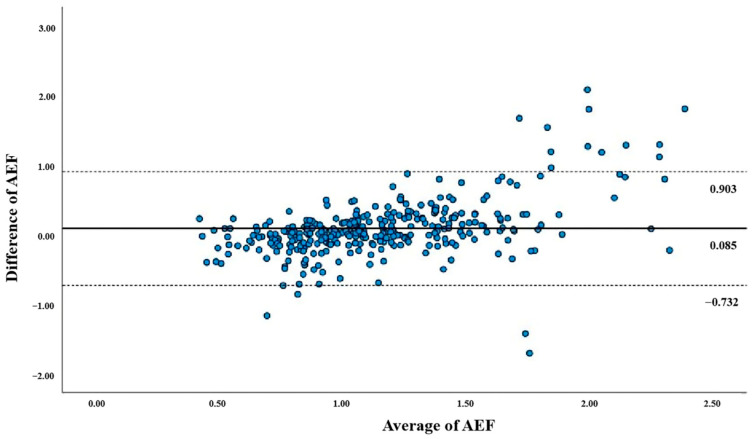
A Bland–Altman plot for AEF_D_ and AEF_S_. The plot demonstrates a small bias (0.085) between AEF_D_ and AEF_S_ (solid lines), with 95% limits of agreement from −0.732 to 0.903 (dotted lines). AEF = arterial enhancement fraction; AEF_S_ = single-energy computed tomography-derived arterial enhancement fraction; AEF_D_ = dual-layer computed tomography-derived arterial enhancement fraction.

The correlation coefficient between the AEF_D_ and AEF_S_ was 0.710 (*p* < 0.001), with a mean inter-method difference of 0.085. Figure 4 shows a scatter plot comparing the AEF_D_ and AEF_S_, while Figure 5 presents a Bland–Altman plot indicating limits of agreement from −0.732 to 0.903.

### 3.4. Diagnostic Efficiency of Spectral Parameters, AEF, and Conventional Image Feature

**Figure 6:**
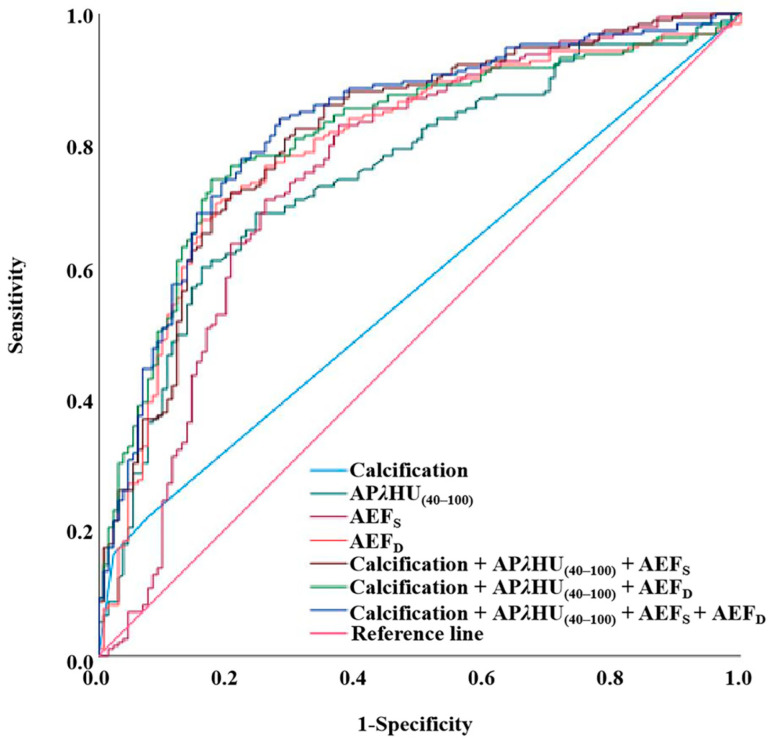
ROC curves comparing the diagnostic performance of individual and combined parameters for malignant micronodules. AEF_D_ alone achieved the highest AUC (0.794) among all single parameters. Its inclusion in multi-parameter sets (with calcification, AEF_S_, and AP*λ*HU_(40–100)_) resulted in the highest overall diagnostic accuracy (AUC = 0.826). ROC = receiver operating characteristic; AP*λ*HU_(40–100)_ = arterial phase slope of the spectral curve (40–100 keV); AEF_S_ = single-energy computed tomography-derived arterial enhancement fraction; AEF_D_ = dual-layer computed tomography-derived arterial enhancement fraction.

The diagnostic efficiency of individual spectral parameters, AEF, conventional imaging features, and their combination is evaluated in Table 4 and illustrated in Figure 6. The AEF_D_ achieved an AUC of 0.794 (95% CI: 0.743–0.845), with a sensitivity of 70.5%, specificity of 81.7%, and accuracy of 75.1%, reducing the unnecessary biopsy rate to 18.3%. AEF_S_ yielded an AUC of 0.753 (95% CI: 0.695–0.810), with a sensitivity of 71.1%, specificity of 74.0%, and accuracy of 72.3%, resulting in an unnecessary biopsy rate of 26.0%. The spectral parameters AP*λ*HU_(40–100)_ showed an AUC of 0.752 (95% CI: 0.698–0.806), with a sensitivity of 68.9%, specificity of 75.6%, and accuracy of 71.7%, leading to an unnecessary biopsy rate of 24.4%. Calcification exhibited an AUC of 0.573 (95% CI: 0.511–0.636), with a sensitivity of 21.6%, specificity of 92.4%, and accuracy of 50.5%, achieving the lowest unnecessary biopsy rate of 7.6%, though with limited clinical utility due to its low sensitivity. Additionally, the performance of multivariable combination was assessed. The combination incorporating calcification, AP*λ*HU_(40–100)_, and AEF_S_ achieved an AUC of 0.811 (95% CI: 0.763–0.860), with a sensitivity of 82.1%, specificity of 69.5%, and accuracy of 74.2%, yielding an unnecessary biopsy rate of 30.5%. The combination incorporating calcification, AP*λ*HU_(40–100)_, and AEF_D_ achieved an AUC of 0.810 (95% CI: 0.762–0.858), with a sensitivity of 74.2%, specificity of 82.4%, and accuracy of 78.4%, demonstrating a superior unnecessary biopsy rate of 17.6%. The comprehensive combination containing calcification, AP*λ*HU_(40–100)_, AEF_S_, and AEF_D_ showed an AUC of 0.826 (95% CI: 0.779–0.872), with a sensitivity of 83.7%, specificity of 71.8%, and accuracy of 78.8%, resulting in an unnecessary biopsy rate of 28.2%.

## 4. Discussion

This study examined how effectively arterial enhancement fraction (AEF) differentiates between thyroid microcarcinoma and benign micronodules. Our findings reveal that AEF greatly improved diagnostic performance. Specially, AEF_D_ had an AUC 0.794, a sensitivity of 70.5%, and an accuracy of 75.1%, surpassing traditional imaging features like calcification. By using straightforward hemodynamic quantification of nodules, this innovative approach establishes AEF as a valuable standalone parameter that enhances the accuracy of diagnosing thyroid micronodules. Moreover, AEF_D_ decreased the rate of unnecessary biopsies, improving preoperative biopsy decision-making.

The quantitative DSCT parameters, such as AP*λ*HU_(40–100)_, APNIC, and APNZ_eff_, showed significant differences between TMC and benign micronodules, consistent with prior studies [27]. Multivariable logistic regression revealed that a lower AP*λ*HU_(40–100)_ value was associated with a higher malignancy probability. Similarly, APNIC was significantly reduced in TMCs, aligning with previous reports [29,30]. This decrease may reflect impaired sodium–iodide symporter function, dysregulated iodine metabolism, reduced uptake, rapid washout, and alterations in the tumor microenvironment that collectively limit iodine accumulation [31]. In contrast, an experimental report contradicts our findings [32], with discrepancies potentially arising from technical factors such as the miscalculation of calcified regions as iodine-rich areas by segmentation algorithms, or measurement bias introduced by intranodular heterogeneity, including hemorrhage or lipid components [33].

AEF_D_, quantifying the arterial-to-venous iodine concentration ratio, effectively differentiates thyroid micronodules. The significantly lower AEF_D_ in malignant nodules reflects their disordered vascular architecture, as documented in Doppler and contrast-enhanced ultrasound studies showing immature, tortuous vasculature with inefficient perfusion [34]. This results in delayed and heterogeneous enhancement on computed tomography (CT), characterized by reduced arterial iodine delivery due to compromised inflow, coupled with increased vascular permeability causing contrast extravasation. Concurrent downregulation of the sodium–iodide symporter further impairs cellular iodine uptake and clearance [31]. These mechanisms collectively lead to relatively elevated venous iodine retention and decreased AEF_D_ in malignancies. Moreover, the strong correlation between AEF_D_ and the hemodynamic parameter AEF_S_ underscores their shared utility in quantifying tumor perfusion, consistent with earlier reports [28].

Our study applied the AEF, specifically AEF_D_ as a novel quantitative functional biomarker that captures tumor-specific hemodynamic abnormalities providing a mechanistic link to malignancy not offered by conventional morphological features. This approach demonstrates satisfactory diagnostic precision for thyroid microcarcinomas by comprehensively capturing tumor heterogeneity and hemodynamic differences between benign and malignant micronodules, outperforming prior strategies reliant on static imaging parameters [27]. AEF_D_ achieved high diagnostic performance as a standalone parameter, with an AUC of 0.794 and specificity of 81.7%, reflecting considerable discriminatory ability and utility in excluding malignancy. Integration of AEF_S_ with spectral parameters and conventional imaging features further improved the AUC to 0.826, sensitivity to 83.7%, and accuracy to 78.8%. Clinically, the high specificity of AEF_D_ (81.7%) significantly reduced unnecessary biopsies, preventing 107 procedures among 131 benign micronodules and lowering the unnecessary biopsy rate to 18.3%. In such borderline cases, a high AEF_D_ value may favor active surveillance, reducing invasive procedures and associated risks, while a low value strengthens the indication for biopsy. Moreover, as it is easily integrated into routine CT protocols, AEF_D_ enhances diagnostic precision and resource efficiency even in resource-limited settings. This study has several limitations. First, its retrospective, single-center design may introduce selection bias and limits the generalizability of our findings. A prospective, multicenter study with a larger and more diverse cohort is needed to validate our results. Second, as most malignancies were papillary thyroid microcarcinomas, the applicability of AEF_D_ to other histological subtypes (e.g., follicular or medullary carcinoma) requires further investigation. Third, manual ROI placement may contribute to inter-observer variability. Future research should focus on leveraging machine learning algorithms to automate ROI segmentation and to develop machine learning-based predictive models. Finally, future correlation of DSCT parameters with molecular biomarkers could facilitate the development of multi-modal diagnostic models, offering deeper insights into tumor biology and further personalizing patient management.

## 5. Conclusions

In conclusion, this study establishes AEF_D_ as an effective imaging biomarker reflecting tumor hemodynamics, demonstrating superior diagnostic performance compared to conventional imaging features for differentiating thyroid microcarcinomas from benign micronodules, while complementing existing quantitative parameters. The application of AEF_D_ may significantly reduce unnecessary biopsy rates, offering a valuable tool for clinical decision-making.

## Figures and Tables

**Table 1:** Clinical characteristics and DSCT parameters between benign and malignant micronodule cohorts.

	Benign Micronodule Cohort (*n* = 131)	Malignant Micronodule Cohort (*n* = 190)	*p* Value
Age, y (%)			<0.001
<50	59 (55.1%)	157 (85.8%)	
≥50	48 (44.9%)	26 (14.2%)	
Gender (%)			0.070
Female	98 (91.6%)	154 (84.2%)	
Male	9 (8.4%)	29 (15.8%)	
Maximum nodule size (mm)	6 (5, 9)	7 (6, 8)	0.392
Calcification (%)			<0.001
Microcalcification	3 (2.3%)	30 (15.8%)	
Macrocalcification	7 (5.3%)	11 (5.8%)	
Absent calcification	121 (92.4%)	149 (78.4%)	
Enhanced blurring (%)			0.722
Yes	45 (34.4%)	124 (65.3%)	
No	86 (65.6%)	66 (34.7%)	
Arterial phase			
*λ*HU_(40–100)_	4.453 (3.928, 5.250)	3.329 (2.707, 4.227)	<0.001
NIC	0.372 (0.319, 0.415)	0.285 (0.227, 0.339)	<0.001
NZ_eff_	0.824 (0.797, 0.843)	0.793 (0.766, 0.814)	<0.001
Venous phase			
*λ*HU_(40–100)_	3.457 (3.046, 4.098)	3.481 (2.930, 4.135)	0.942
NIC	0.680 (0.627, 0.771)	0.720 (0.609, 0.842)	0.194
NZ_eff_	0.943 (0.929, 0.958)	0.952 (0.927, 0.973)	0.103

DSCT = dual-layer spectral computed tomography; *λ*HU_(40–100)_ = slope of the spectral curve (40–100 keV); NIC = normalized iodine concentration; NZ_eff_ = normalized effective atomic number.

**Table 2:** Multivariate logistic regression analyses results for quantitative parameters.

Variables	Coefficient (*β*)	SD	Wald	Odds (95% CI)	*p* Value	VIF	F Statistics	Standardized *R*^2^
							22.752	0.177
AP*λ*HU_(40–100)_	0.511	0.161	10.003	0.600 (0.437, 0.823)	0.002	2.356		
APNIC	4.589	3.126	2.154	0.010 (0.000, 4.658)	0.142	3.933		
APNZ_eff_	0.883	4.755	0.034	0.414 (0.000, 4613.544)	0.853	2.231		

AP*λ*HU_(40–100)_ = arterial phase slope of the spectral curve (40–100 keV); APNIC = arterial phase normalized iodine concentration; APNZ_eff_ = arterial phase normalized effective atomic number; *β =* regression coefficient; SD = standard deviation; CI = confidence interval; VIF = variance inflation factor.

**Table 3:** AEF_S_ and AEF_D_ between benign and malignant micronodule cohorts.

AEF Value	Benign Micronodule Cohorts (*n* = 131)	Malignant Micronodule Cohorts (*n* = 190)	*p* Value
AEF_S_	1.436 (1.126, 1.697)	0.964 (0.747, 1.210)	<0.001
AEF_D_	1.259 (1.112, 1.469)	0.958 (0.811, 1.123)	<0.001

AEF = arterial enhancement fraction; AEF_S_ = single-energy computed tomography-derived arterial enhancement fraction; AEF_D_ = dual-layer computed tomography-derived arterial enhancement fraction.

**Table 4:** Diagnostic efficiency of individual and combined parameters for diagnosing malignant micronodules.

	AUC (95% CI)	Unnecessary Biopsy Rate (%)	Sensitivity (%)	Specificity (%)	Accuracy (%)	PPV (%)	NPV (%)
Calcification	0.573 (0.511, 0.636)	7.6% (10/131)	21.6%	92.4%	50.5%	80.4%	44.8%
AP*λ*HU_(40–100)_	0.752 (0.698, 0.806)	24.4% (32/131)	68.9%	75.6%	71.7%	80.4%	62.7%
AEF_S_	0.753 (0.695, 0.810)	26.0% (34/131)	71.1%	74.0%	72.3%	79.9%	63.8%
AEF_D_	0.794 (0.743, 0.845)	18.3% (24/131)	70.5%	81.7%	75.1%	84.8%	65.6%
Calcification + AP*λ*HU_(40–100)_ + AEF_S_	0.811 (0.763, 0.860)	30.5% (40/131)	82.1%	69.5%	74.2%	79.6%	72.8%
Calcification + APλHU_(40–100)_ + AEF_D_	0.810 (0.762, 0.858)	17.6% (23/131)	74.2%	82.4%	78.4%	86.0%	68.8%
Calcification + AP*λ*HU_(40–100)_ + AEF_S_ + AEF_D_	0.826 (0.779, 0.872)	28.2% (37/131)	83.7%	71.8%	78.8%	81.1%	75.2%

AUC = area under the curve; CI = confidence interval; PPV = positive predictive value; NPV = negative predictive value; AP*λ*HU_(40–100)_ = arterial phase slope of the spectral curve (40–100 keV); AEF_S_ = single-energy computed tomography-derived arterial enhancement fraction; AEF_D_ = dual-layer computed tomography-derived arterial enhancement fraction.

## Data Availability

The data are not publicly available due to legal restrictions but can be obtained from the corresponding author upon reasonable request.

## Abbreviations

The following abbreviations are used in this manuscript:

DSCT	Dual-layer spectral computed tomography
AEF	Arterial enhancement fraction
NIC	Normalized iodine concentration
NZ_eff_	Normalized effective atomic number
*λ*HU	Slope of spectral Hounsfield unit curve
SECT	Single-energy computed tomography
AEF_S_	SECT-derived AEF
AEF_D_	DSCT-derived AEF
AUC	Area under the curve
ROC	Receiver operating characteristic
OR	Odds ratio
TMC	Thyroid microcarcinoma
FNAC	Fine needle aspiration cytology
^F^18-FDG-PET	18-fludeoxyglucose positron emission tomography
CT	Computed tomography
MRI	Magnetic resonance imaging
FOV	Field of view
ROI	Region of interest
AP	Arterial phase
VP	Venous phase
IC	Iodine concentration
Z_eff_	Effective atomic number
HU	Hounsfield unit
IQR	Interquartile range
SD	Standard deviation
VIF	Variance inflation factor
CI	Confidence interval
PPV	Positive predictive value
NPV	Negative predictive value
